# LC-MS-based metabolite profiling of aqueous extract of *Pergularia tomentosa* L. and its anti-hyperglycemic effect 

**DOI:** 10.22038/IJBMS.2022.65646.14441

**Published:** 2022-12

**Authors:** Seyed Hamzeh Hosseini, Fereshteh Ezzati Ghadi, Abdollah Ramzani Ghara, Antonietta Cerulli, Abolfazl Shakeri, Sonia Piacente

**Affiliations:** 1 Department of Biology, Faculty of Science, University of Jiroft, Jiroft, Iran; 2 Dipartimento di Farmacia, Università degli Studi di Salerno, Via Giovanni Paolo II 132, 84084 Fisciano, Salerno, Italy; 3 Department of Pharmacognosy, School of Pharmacy, Mashhad University of Medical Sciences, Mashhad, Iran

**Keywords:** Cardenolides, Diabetes mellitus, LC-MS, Metabolite profiling, *Pergularia tomentosa*, Phytochemical

## Abstract

**Objective(s):**

In this study, to find scientific evidence for the traditional use of *Pergularia tomentosa* as an anti-diabetic remedy, the effects of its aqueous extract on streptozotocin-induced diabetes mellitus in rats were evaluated.

**Materials and Methods:**

Wistar rats were fasted overnight and diabetes mellitus was induced using streptozotocin (50 mg/kg body weight). The rats were randomly and equally divided into four groups (n=5): group I (normoglycaemic control), group II (diabetic rats), group III (diabetic rats treated with 200 mg/kg BW of an aqueous extract of *P. tomentosa*), group IV (normoglycemic rats treated with 200 mg/kg BW of an aqueous extract of *P. tomentosa*). Chemical profiling of the aqueous extract was carried out using liquid chromatography coupled with electrospray ionization and multiple-stage linear ion-trap and orbitrap high-resolution mass spectrometry (LC-ESI/LTQOrbitrap/MS/MS). In addition, the quantitative determination of the main cardenolides in the extract was carried out by an analytical approach based on LC coupled to tandem mass spectrometry with ESI source and hybrid triple quadrupole-linear ion trap mass analyzer (LC-ESI/QTrap/MS/MS).

**Results:**

Aqueous extract of *P. tomentosa* showed a reasonable reduction in blood glucose level. Probably, the *P. tomentosa* effect on hyperglycemic and hyperlipidemic diabetic animals was associated with antioxidant properties, triglyceride levels, as well as liver enzymes. Meanwhile, LC-ESI/LTQOrbitrap/MS/MS analysis led to identification of double-linked cardenolides along with cardenolides and flavone glycosides as the main bioactive compounds.

**Conclusion:**

The extract decreased the glucose level and induced a beneficial effect on the lipid profile, probably due to the presence of cardenolide glycosides.

## Introduction

Diabetes is a metabolic disorder characterized by persistent hyperglycemia and dysfunctional metabolism of carbohydrates, fats, and protein metabolism (1, 2). World Health Organization (WHO) has classified this pathology as type-1 diabetes (β-cell destruction), type-2 diabetes (insulin resistance with insulin hypo-secretion), gestational diabetes, and other specific kinds of diabetes including genetic defect in β-cell-function, disease of the pancreas and chemical/drug-induced diabetes (3, 4). 

In particular, in type 2 diabetes mellitus, the body cells do not use insulin effectively, and consequently, the glucose level goes up. Such a deficiency results in an increased concentration of glucose in the blood, which in turn damages many of the body systems, in particular, blood vessels and nerves (5). 

Hyperglycemia is one of the major characteristics of diabetes, which can lead to serious complications. It may be due to impairment in the equilibrium between ROS and antioxidant defense capacity (6). There are several reports that are attriin which hyperglycemia (the blood sugar level >120 mg/dl) is considered diabetes (7-9). Chronic hyperglycemia is associated with the development of diabetic complications. Several signaling pathways can be altered by having hyperglycemia in different tissues, producing oxidative stress, the formation of advanced glycation end products (AGEs), as well as the secretion of the pro-inflammatory cytokines and cellular death (pathological autophagy and/or apoptosis). In particular, oxidative stress has an important role in the progression of diabetic complications and could culminate in tissue injuries in the heart, retina, kidneys, and the nervous system (10, 11).

The pharmacological agents, used currently for the treatment of diabetes mellitus include mainly oral anti-diabetic drugs as well as insulin subcutaneous therapy. These treatments are used as monotherapy or in different combinations to control diabetic conditions. Some issues, however, limit the effectiveness of these options, such as failure to hinder diabetic complications and prominent side effects (12, 13). WHO reported that 366 million people would be affected by type 2 diabetes mellitus by 2030, increasing the risk of morbidity and mortality due to cardiovascular disease (14). Therefore, there is a growing interest in investigating medicinal plants recognized in Traditional Medicine as a remedy to treat diabetes. Some plants of the Asclepiadaceae family, *Calotropis procera *and *Pergularia daemia,* have been reported to exert *in vivo* anti-diabetic activity (15). *Pergularia tomentosa* L. is a 50 cm high wild perennial shrub, native of the Middle East and North Africa, known in Iran as Labashir or Keshtuk*.* *P. tomentosa* displays a lot of secondary metabolites responsible for biological activities in the different parts of the plant. Previous investigations on *P. tomentosa* highlighted the occurrence of cardiac glycosides including desglucouzarin, coroglaucigenin, and uzarigenin in the leaves (13, 16); uzarigenin, ghalakinoside, calactin, 6′-hydroxycalactin,6′-hydroxy-16α-acetoxycalactin, 16α-hydroxycalactin, 12′-dehydroxyghalakinoside, 3-*O*-β-glucopyranosiylcalactin, and 6′-dehydroxyghalakinoside in the roots (17, 18). Moreover, our recent publications reported isolation and identification of double-linked cardenolides and flavonol glycosides from the aerial parts of *P. Tomentosa *(19, 20). Chemically, cardiac glycosides are compounds characterized by a steroidal nucleus with a lactone moiety at position 17 which leads to the chemical classification of subfamilies as cardenolides or bufadienolides with an unsaturated butyrolactone and α-pyrone ring, respectively; the steroidal skeleton links at position 3 is a sugar chain. In cardiac glycosides produced by plants of the *Asclepiadaceae* family, the A/B rings are transfused, resulting thus in rather flat structures. The above-mentioned fusion gives the aglycon nucleus of these cardiac glycosides a typical “U” shape, resulting in a markedly more potent binding to Na^+^/K^+^-ATPase pump (particularly to Na^+^/K^+^-ATPase α1 subunits) (18). Different parts of *P. tomentosa* have been reported to exert molluscicidal activity (20), cause apoptotic cell death of Kaposi’s sarcoma cells (17), possess autophagic properties (22) to prevent bronchitis, constipation and skin diseases, and to exert hypoglycemic effects (21, 23).

Hyphenated techniques are playing increasingly important roles in support of phytochemical investigations, and in particular, high liquid chromatography (HPLC) coupled with mass spectrometry (MS) is considered a powerful tool to define the metabolite profiling of plant extracts. In this work, with the aim to correlate the hypoglycemic activity to the chemical composition, a qualitative study of *P. tomentosa* aerial parts was carried out by using high-performance liquid chromatography coupled to electrospray negative ionization Orbitrap multicollisional high-resolution mass spectrometry (LC-ESI/LTQOrbitrap/MS/MS). In particular, the Linear Trap Quadrupole-Orbitrap MS analyzer was used since it has MS^n ^capabilities for enhanced levels of structural analysis (24, 25). In this way, the occurrence of a wide range of cardenolides along with flavonoids has been highlighted. Moreover, the quantitative determination of the main cardenolides in the aqueous extract of *P. tomentosa* aerial parts was carried out by an analytical approach based on LC-ESI/QTrap/MS/MS, using a very sensitive and selective mass tandem experiment called Multiple Reaction Monitoring (MRM).

## Materials and Methods

***Chemicals***

Streptozotocin (STZ) was purchased from Sigma. All other chemicals and reagents used were of analytical grade. Acetonitrile, formic acid, and water for LC-MS were bought from Merck (Merck KGaA, Darmstadt, Germany).

***General procedures***

HRESIMS spectra were carried out using an LTQ Orbitrap XL mass spectrometer (Thermo Fisher Scientific, San Jose, CA, USA) operating in negative ion mode. The Orbitrap mass analyzer was calibrated according to the manufacturer’s directions using a mixture of caffeine, methionine-arginine-phenylalanine-alanine-acetate (MRFA), sodium dodecyl sulfate, sodium taurocholate, and Ultramark 1621. Data were collected and analyzed using the software (Xcalibur) provided by the manufacturer.

***Plant material***

Fresh aerial parts of *P. tomentosa* L. were collected in Kahnouj, Kerman province, Iran, in October 2019 and identified by Mr. Ahmad Pormirzaee. Plant material was air-dried and stored at room temperature. The voucher specimen (no. 8644) was deposited at the Herbarium of the Kerman Agricultural & Natural Resources Research & Education Center, Kerman, Iran.

The aerial parts of *P. tomentosa* L., finely powdered using a mechanical grinder, were air-dried and subjected to extraction. 500 g of powder was suspended in distilled water (2.000 l) for 24 hr at room temperature. The suspended plant material was frequently shaken and then filtered. The process was repeated three times. The aqueous extract was submitted to lyophilization to remove water, in this way 7.00 g of lyophilized aqueous extract was obtained.

The aqueous extract of *P. tomentosa* L. aerial parts has been used for biological assays and has been submitted to LC-MS to obtain the metabolite profile.

***Animals***

Experiments were performed on twenty male Wistar rats (11 weeks old) weighing 180-220 g (Pasteur Institute of Kerman, Iran). All animal experiments were performed in accordance with the UK Animals (Scientific Procedures) Act 1986 and associated guidelines.

***Acute toxicity study***

Determination of acute toxicity of the extract was performed by using the OECD (Organization of Economic Co-operation and Development) guidelines for testing chemicals (Acute Oral Toxicity- section 423). To three male Wistar rats, single doses (200 mg/kg body weight) of the aqueous extract of *P. tomentosa* aerial parts were administered orally, after overnight fasting. Rats were observed for symptoms and weighed at post-administration intervals of 1, 3, and 4 hr and then twice per day for the subsequent 14 days (26).

***Induction of diabetes***

Animals were housed with a 12 hr light-dark cycle with free access to food and water. Intraperitoneal injection of STZ (50 mg/kg body weight) in 0.1 M citrate buffer (pH 4.5) was performed to induce diabetes mellitus (type I). Rats were fasted for 12-14 hr before the induction of experimental diabetes. Diabetes was confirmed by evaluation of levels of serum glucose after 42 hr (above 120 mg/dl) (7). The animals were treated with *P. tomentosa *extract by oral gavage for 15 days. All experimental protocols were approved by the University of Jiroft (3818-97-3), Jiroft, Iran. Animals were randomly and equally divided into four groups (n=5).

Experimental design was as follows:

Group I- control rats

Group II- rats treated with STZ 

Group III- diabetic rats treated with *P.* *tomentosa *extract (200 mg/kg body weight).

Group IV- normal rats treated with *P.* *tomentosa *extract (200 mg/kg body weight)

At the end of the study, blood samples of all fasted rats (12-14 hr) were collected for biochemical analysis. 

***Biochemical parameters***

At the end of the treatment period, the rats were anesthetized with diethyl ether and blood samples were collected by cardiac puncture. The serum levels of ALT, AST, LDL, HDL, TG, MDA, and blood glucose were measured using the automated chemistry analyzer (Auto-analyzer, Hitachi 912) and spectrophotometer.

***Histological examination***

Pancreas tissue was fixed in 10% neutral buffered formalin. After the pancreas was sectioned into 5 μm thick slices, and stained with hematoxylin/eosin for histological examination.

***LC-ESI/LTQOrbitrap/MS/MS analysis***

The qualitative LC-MS profile of the aqueous extract of *P. tomentosa* aerial parts was obtained by LC-ESI/LTQOrbitrap/MS/MS analysis. A quaternary Accela 600 pump and an Accela autosampler coupled to an LTQOrbitrap XL (ThermoScientific, San Jose, CA, USA), operating in the negative electrospray ionization mode were used.

The separation was carried out using a C18 reversed-phase (RP) column (2.1 x 250 mm; X-Terra MS C18 5 µm; Waters, Milford, MA, USA) at a flow rate of 0.2 ml/min and coupled to an LTQ-Orbitrap XL mass spectrometer. Linear gradient elution was acquired by using water with 0.1% formic acid as eluent A and acetonitrile with 0.1% formic acid as B. 

The HPLC gradient was set to 10% B at 0 min and then increased to 60% B from 0 to 25 min, followed by an increase to 100% B from 25 to 35 min, holding it for 10 min, before returning to the starting percentage. The mass range was from 200 to 1200 *m/z* with a resolution of 30000. The *m/z* of each identified compound was calculated to 4 decimal places and measured with a mass accuracy of <3 ppm. The source voltage was 3.5 kV, the capillary voltage -48 kV, the tube lens offset -176.5 V, and the capillary temperature was set at 280 ^°^C, the auxiliary gas was set at 5 (arbitrary units), and the sheath gas at 15 (arbitrary units). In full LC-ESI-MS experiments, the Total Ion Current (TIC) profile was produced by monitoring the intensity of all the ions produced and acquired in every scan during the chromatographic run. In order to get structural information, Data dependent experiments were performed by acquiring MS2 spectra of the most intense ions produced during the acquisition; a normalization collision energy at 30%, a minimum signal threshold at 250, an isolation width at 2.0, and multiple-stage tandem mass have been used (20). For the aqueous extract of *P. tomentosa* aerial parts, the autosampler was set to inject 2 μl of extract (0.5 mg/ml); for standards solution, the autosampler was set to inject 2 μl of each standard (1.0 mg/ml).

***LC-ESI/QTrap/MS/MS analysis***

Quantitative analysis was performed on an LC-ESI/QTrap/MS system, operating in Multiple Reaction Monitoring (MRM) mode, and separation was performed on C18 reversed-phase (RP) column (1.5 x 2.00 mm); Luna MS C18 5 µm; Phenomenex, Aschaffenburg, Germany) kept at 30 ^°^C, a flow rate of 0.23 ml/min, and a mobile phase consisting of a combination of A (0.1% HCOOH in water, v/v) and B (0.1% HCOOH in acetonitrile, v/v). The HPLC gradient used was set to first a 0 min with 10% B which was then increased to 60% B from 0 to 25 min, followed by an increase to 100% B from 25 to 30 min, holding it for 5 min. before returning to the starting percentage.

***Calibration and quantification***

Stock solutions (1 mg/ml) of isolated compounds used as external standards (ES) were prepared by dissolving each compound in a solution of methanol/water (70:30 v/v) (18). Calibration curves were constructed by injecting 4 µl of each standard solution at each concentration level in triplicate. Linear regression analysis was performed using the Analyst 1.6.2 Software provided by the manufacturer (AB Sciex). 

In order to validate the LC-ESI/QTrap/MS/MS method, precision (at six concentrations for each compound: 0.2, 0.5, 1.0, 2.5, 5, 10, µg/ml), specificity, linear range, limit of detection (LOD), and limit of quantification (LOQ) were evaluated. LOD and LOQ for each target standard compound were determined, under the optimized conditions, by the serial dilution of a standard solution until the signal-to-noise ratios (S/Ns) were 3:1 and 10:1, respectively (27). The LOD for each analyte was from 0.054 to 0.093 µg/µl and LOQ from 0.18 to 0.31 µg/µl.

***Statistical analysis***

The statistical analysis was performed using one-way ANOVA (analysis of variance). The *post hoc* method used in statistical analysis was LSD (Least Significant Difference) test. A difference in the mean values of *P*<0.05 was considered to be statistically significant.

## Results

In the present study, the rats were anesthetized with diethyl ether, and immediately the blood samples were collected for blood analysis. A previous study showed that diethyl ether may not result in both hyper and hypoglycemia when used as an anesthetic agent (28). The effects of *P. tomentosa* extract on water intake, body weight, and blood glucose levels in diabetic rats are shown in Table 1. In particular, after injection of STZ, a significant loss (*P*<0.001) in the final body weight if compared with the initial body weight was observed. In the diabetic group treated with *P. tomentosa*, the final body weight was higher than the initial body weight. 

In the current study, blood samples of all fasted rats (12-14 hr) were collected and blood sugar level above 120 mg/dl was considered. Oral administration of *P. tomentosa* (Table 1) caused significant declines in the fasting blood glucose of diabetic rats compared with untreated diabetic rats. A significant difference between diabetic rats and diabetic rats treated with *P. tomentosa *has been observed. Moreover, the administration of *P. tomentosa, *at a dose of 200 mg/kg to diabetic rats, caused a significant (*P*<0.01) reduction of blood glucose levels and water intake.

***Influence of P. tomentosa aqueous extract on lipids blood level***

Results of lipids blood level were shown in Table 2. In the present study, an increase in triglyceride levels after injection of STZ was observed. The triglyceride level of the diabetic group was significantly higher than the normal control, while the administration of *P. tomentosa *to STZ-treated diabetic rats, caused a significant reduction of blood triglyceride levels. Moreover, HDL levels were decreased in diabetic rats, while *P. tomentosa* administration to diabetic rats increased HDL concentration. LDL levels did not show evident significant differences between tested groups. Moreover, the MDA level was increased (*P*<0.001) in the diabetic group and in diabetic rats treated with *P. tomentosa* if compared with the control group (Table 2). ALT and AST levels were shown in Table 2. Blood levels of ALT and AST were increased in the STZ-treated rats. Whereas, *P. tomentosa * administration to the STZ-treated rats decreased the ALT and AST levels.

***Histological assessment of pancreas, kidney, and liver by Hematoxylin and Eosin staining***

To evaluate the effects of *P. tomentosa *aqueous extract on the pancreas, kidney, and liver of diabetic and control rats, the histological analysis of the above-mentioned organs has been performed.

**Figure 1:**
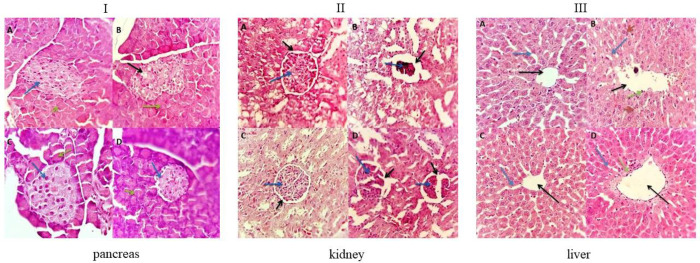
Effect of *Pergularia tomentos*a extract on the histological profile of pancreas (I), kidney (II), and liver (III) in different treated groups of Control rats. I) Islet β-cells (black arrow) and exocrine components (green arrow), II) glomerular capillary (blue arrow), urinary space (black arrow), and III) central vine (black arrow), sinusoids (blue arrow), binucleated hepatocytes (red arrow) and Kupffer cells (green arrow)

The pancreas cells of control rats showed normal acinar cells that stained strongly and arranged in lobules with prominent nuclei. As shown in Figure 1-IA, in control rats, the islet cells were embedded within the acinar cells and surrounded by a capsule. The pancreas of the diabetic untreated group revealed a high level of cellular damage; in particular, diabetic rats revealed pathological changes in both exocrine and endocrine components. Islet β-cells were almost entirely lost in STZ-treated rats. Islets of Langerhans showed hyaline and necrotic changes (black arrow). Wider interlobular and intralobular ducts were observed (Figure 1-IB). *P. tomentosa* administration to diabetic rats determined marked improvement of the cell injury, as evident from the partial restoration of islets of Langerhans and exocrine components (green arrow) (Figure 1-ID). The pancreas of control rats treated with *P. tomentosa* showed normal architecture of the pancreas (Figure 1-IC).

The kidney section of the control untreated group and *P. tomentosa* treated group showed normal architecture of glomerular capillary (blue arrow), glomerular tubule, and urinary space (black arrow), with normal basement membrane and capillaries (Figure 1-IIA and IIC). In diabetic rats (Figure 1-IIB), kidney sections showed mild thickening of the basement membrane, and atrophy of glomerular capillaries (blue arrow), with increased Bowman’s space (urinary space, black arrow). Diabetic rats treated with *P. tomentosa* showed features of healing, i.e., normal structure of basal membrane and glomerulus. Moreover, Bowman’s space was improved toward normal condition after treatment with *P. tomentosa* (Figure 1-IID). Therefore, the kidneys of diabetic rats treated with *P. tomentosa* showed an improvement if compared with those of the diabetic untreated group. In detail, the extract slowed down the renal impairment associated with diabetes mellitus. 

The liver of control rats (Figure 1-IIIA) showed a preserved architecture with central vine (black arrow), hepatocytes, and sinusoids (blue arrow). Moreover, normal sinusoids with flatted endothelial cells were seen (Figure 1-IIIA and IIIC). The histology of liver sections obtained from diabetic rats showed loss of the normal architecture with congested central vein (black arrow), disarranged sinusoids (blue arrow), binucleated hepatocytes (red arrow), and more Kupffer cells (green arrow) (Figure 1-IIIB). Liver sections of diabetic rats treated with *P. tomentosa* (Figure 1-IIID) showed almost normal liver histology with slightly dilated sinusoids and a lesser degree of inflammation.

***LC-MS analysis of specialized metabolites occurring in aqueous extract of P. tomentosa aerial parts***

In order to correlate the hypoglycemic activity to the chemical composition, the aqueous extract of *P. tomentosa* aerial parts has been investigated by an analytical approach based on LC-ESI/LTQOrbitrap/MS/MS, operating in the same conditions reported previously (19). LC-MS analysis did not highlight any interesting compound under *m/z* 200, consequently, the range *m/z* 200-1200 was selected. The analysis of LC-HRMS spectra allowed us to assign both accurate molecular mass and molecular formula to the [M-H]^-^ pseudomolecular ions occurring in the LC-MS profile. Identification of compounds has been performed based on the retention times, accurate masses, and characteristic fragmentation patterns, and by comparison with literature data on *P. tomentosa *(19). Moreover, to unambiguously establish the molecular structure of compounds **1-23**, and to discriminate among structural isomers or stereoisomers, LC-MS analysis of naturally occurring standards, previously isolated from the aerial parts and roots of *P. tomentosa*, has been carried out. 

**Figure 2:**
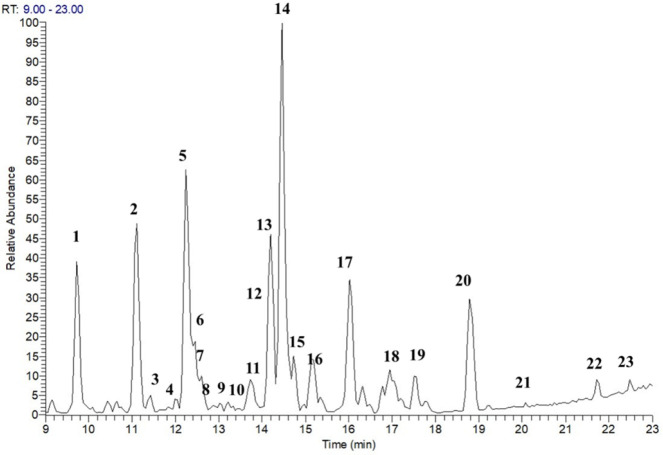
ESI/LTQOrbitrap/MS profile (negative-ion mode) of the aqueous extract of *Pergularia tomentos*a aerial parts

**Figure 3:**
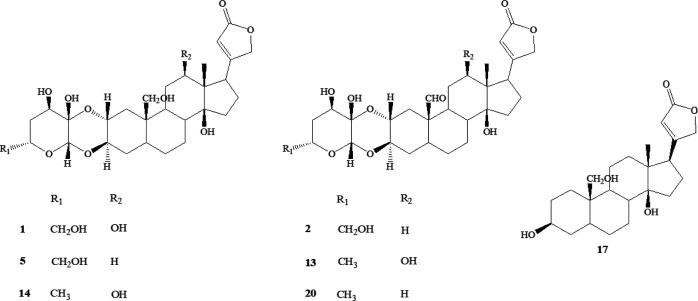
Chemical structures of the main cardenolides occurring in the aqueous extract of *Pergularia tomentos*a aerial parts

In this way, 23 metabolites could be identified. In particular, the LC-MS analysis of *P. tomentosa* extract suggested the occurrence of cardenolides (4**, **8**, **16**-**17**, **21), doubly linked cardenolides (1**-**3**, **5**, **7**, **12**-**15**, **18**-**20**, **22**-**23), and flavone glycosides (6**, **9**-**11) (Figures 2 and 3, Table 3). 

LC-MS analysis showed some compounds with the same pseudomolecular ions; this is the case of compounds **2**, **7**, 12, and 13 with a pseudomolecular ion [(M+HCOOH)-H]^-^ at *m/z* 593, compounds **5** and **14** with a pseudomolecular ion [(M+HCOOH)-H]^-^ at *m/z* 595, as well as compounds **19** and **20** with a pseudomolecular ion [(M+HCOOH)-H]^-^ at *m/z* 577 (Table 3). 

***Quantitative Analysis by LC-ESI/QTrap/MS/MS of the main cardenolides occurring in the aqueous extract of P. tomentosa aerial parts***

For the most representative compounds, ghalakinoside (1), 6′-hydroxycalactin (2), 12′-dehydroxyghalakinoside (5), 12β-hydroxycalactin (13), 6′-dehydroxyghalakinoside (14), coroglaucigenin (17), and calactin (20) (Figure 3), an LC-ESI/QTrap/MS analysis, using MRM mode, has been carried out. In detail, MRM (Multiple Reaction Monitoring) is a tandem mass spectrometric technique in which to determine the amount of selected metabolites a specific transition from a precursor ion to a product ion is monitored for each compound, assuring a very high selectivity and sensitivity. Based on the fragmentation pattern shown by each isolated compound used as standard in ESI/MS/MS spectrum, MRM transitions have been chosen (Table 4). In particular, the [M-H]^-^ pseudomolecular ion at *m/z *611, corresponding to ghalakinoside (1), showed the main product ion [(M-190)-H]^-^ at *m/z* 421, due to the loss of formic acid and neutral sugar unit at position C-3. 

Negative LC-MS spectra of compounds **2**,** 5**,** 13**,** 14**, **17**, and **20** were characterized by molecular [(M+FA)-H]^-^ anions formed as adducts with formic acid; for this reason for [M-H]^-^ pseudomolecular ion at *m/z* 593 (2, 13), *m/z* 595 (5, 14), *m/z* 435 (17), and *m/z* 577 (20), the transition to *m/z* 547, 549, 389, and 531, respectively, originated by the neutral loss of the formic group (46 Da), was chosen for MRM analysis. 

## Discussion

Based on the hypoglycemic activity reported for plants belonging to the *Asclepiadaceae* family, the anti-diabetic activity of an aqueous extract of* P. tomentosa* aerial parts in rats affected by streptozotocin-induced diabetes has been evaluated. 

The influence of an aqueous extract of *P. tomentosa* aerial parts on body weights and blood glucose levels were evaluated by *in vivo* assays. Moreover, the level of alanine and aspartate transaminase (ALT and AST), and lipid peroxidation in the serum of rats as well as histopathological studies have been determined.

 In the present study, STZ decreased the body weight while *P. tomentosa *administration to diabetic rats increased the final body weights. Probably, the improvement in body weight could be associated with positive modification of blood sugar, which enhanced weight gain through successful glucose utilization (29-31). 

Group 4 was designed to investigate the possible side effects of the aqueous extract of *P. tomentosa * on non-diabetic rats (32). Results showed there was no difference between Group 4 and normal rats. 

Results showed that blood sugar levels were significantly increased in diabetic rats but *P. tomentosa *supplementation to the STZ group decreased the blood sugar if compared with normal rats. These results are in agreement with previous studies (33).

STZ caused damage to pancreas β-cells, leading to diabetes mellitus (34). Antioxidants play an important role in the prevention and treatment of diabetes due to scavenging various reactive oxygen species (35, 36).

***Influence of P. tomentosa aqueous extract on lipids blood level***

In the present study, an increase in triglyceride levels after injection of STZ was observed compared with normal rats. Hyperlipidemia is a known complication of diabetes (37). Elevated plasma triglyceride levels in type 1 diabetes, is due to its over-production or underutilization (32). The obtained results are in agreement with the literature reporting that the insulin action on lipoprotein metabolism is exerted mainly through the lipolysis increase of triglyceride-rich lipoproteins by stimulating lipase and lipolysis prevention of fats stored in tissues by inhibition of hormone-sensitive lipase (38).

Diabetic rats treated with the aqueous extract of *P. tomentosa* aerial parts displayed triglyceride levels not significantly different from the normal control. The elevation of triglycerides and reduced HDL levels are the main features of dyslipidemia in diabetics. Moreover, diabetic rats showed a significant decrease in plasma HDL levels. While treatment with *P. tomentosa * kept the values of HDL, near normal values which is in agreement with the previous study (33). The decrease in the HDL levels may be due to increased LDL or/deficiency in lecithin cholesterol acyltransferase (39).

Furthermore, a decrease of ALT and AST levels in the serum of the diabetic rats treated with *P. tomentosa* extract, probably due to the compounds exerting free radical scavenging activity protecting liver cells against lipid peroxidation, was observed.

From the histopathology of the liver, an improvement of *P. tomentosa* treated diabetic rats was observed. The liver of the diabetic untreated group showed evidence of congestion, inflammation, and necrosis. These observations suggested steatosis of the liver and evidenced the toxic effects of STZ on the liver of rats (40). In detail, treatment with *P. tomentosa* extract improved the hepatic architecture; moreover, as evidenced by the liver biochemical parameters, a hepatoprotective effect could be attributed to *P. tomentosa* extract. These observations showed that the extract could confer some protective effect on the pancreas, consequently improving glucose metabolism. This could be attributed to the antioxidant effect of some of the phytochemicals in *P. tomentosa* which prevent streptozotocin-induced free radical destruction of the pancreatic islets.

***LC-MS qualitative profile of aqueous extract of P. tomentosa aerial parts***

The careful analysis of LC-ESI/LTQOrbitrap/MS/MS of aqueous extract of *P. tomentosa* aerial parts allowed to highlight the occurrence of specialized metabolites belonging to flavonoids glycosides and cardiac glycosides. The analysis of the LC-MS profile demonstrated the occurrence of cardenolides. In particular, this investigation confirmed that the aerial parts of *P. tomentosa* represent a source of doubly-linked cardenolides. Chemically, they can be considered as belonging to two groups: the calactin derivatives (1, 2, 5, 13, 14, 15, and 20) and the calotropin derivatives (3, 7, 12, 18, 19, 22, and 23), differing in the configuration at C-3′. The doubly-linked cardenolides have been found in the Asclepiadaceae family, particularly in the genera *Asclepias*, *Calotropis*, and *Pergularia*. This structural feature is rarely found in the cardenolides from other families. Moreover, doubly-linked cardenolides represent a group of secondary metabolites that share the capacity to bind the extracellular surface of the main ion transport protein in the cell, the membrane-inserted sodium pump (Na^+^/K^+^-ATPase) (18). Earlier studies showed the hypoglycemic effect exe

rted by ouabain, a cardiac glycoside present in plants belonging to the Asclepiadaceae family; in particular, this compound displayed a significant decrease in glucose and glycerol concentrations (41, 42).

***Quantitative analysis of the main cardenolides in the aqueous extract of P. tomentosa aerial parts***

For the most representative compounds, an LC-ESI/QTrap/MS/MS analysis, using MRM mode, has been carried out. Based on the transitions selected for MRM experiments, the amount (mg/100 g dry weight) of main cardenolides in the aqueous extract of *P. tomentosa* aerial parts (Table 4) was determined. 6′-dehydroxyghalakinoside (14) exhibited the highest concentration (11.60 mg/100 g) followed by 12′-dehydroxyghalakinoside (5) (7.11 mg/ 100 g); the other analyzed compounds occurred in the extract in the concentration range 0.95–5.58 mg/100 g of dry aerial parts.

**Table 1:** Effect of *Pergularia tomentos*a extract on body weight, water intake, and blood glucose of STZ-induced diabetes in rats

**Group**	** Body weight**			**Blood Glucose (mg/dl) Water intake (mL)**	
	Initial body weight	Second body weight	Final body weight		
**Control**	221.50 ± 26.43	269.75 ± 15.58	271.575 ± 16.2	111.00 ± 18.85	180.00 ± 24.49
**Diabetic untreated group**	217± 11.94	227 ± 10.42^c^	207.93 ± 17.11^c^	518.50 ± 118.40^c^	617.50 ± 62.38^ c^
**Diabetic treated (** ***P. tomentosa*** **)** **group**	228 ± 5.88	264 ± 7.02^z^	249.00 ± 14.63^y^	126.00 ± 43.87^z^	257.50 ± 28.72 ^a, z^
**Treated (** ***P. tomentosa*** **) group**	215.75 ± 4.34	269 ± 15.52	265.92 ± 18.78	95.25 ± 10.11	187.50 ± 25.00

Results are presented as mean±SD. n=5. ^c^ *P<*0.001 represents the statistically significant difference between the control and all treated groups

^y^ *P<*0.001 and ^z^ *P<*0.001 also show a significant difference between the diabetic and diabetic group treated with *P. Tomentosa*

A) Control group. B) Diabetic rats treated with STZ. C) *P. tomentosa* treated rats. D) Diabetic rats treated with *P. tomentosa* (Hematoxylin-Eosin staining, magnification ×400)

**Table 2:** Effects of *Pergularia tomentos*a extract on triglyceride, HDL LDL, AST, ALT, and serum lipid peroxidation on serum of control and all treated rats

Group	Triglyceride (mg/dl)	HDL (mg/dl)	LDL (mg/dl)	AST (IU/L)	ALT (IU/L)	Lipid peroxidation (nmoles of MDA/ min/ mg protein)
Control	89.33 ± 13.05	52.00 ± 1.41	7.66 ± 1.52	85.50 ± 9.19	61.00 ± 5.65	0.185 ± 0.005
Diabetic untreated group	142.25 ± 27.03^a^	37.33 ± 3.78^a^	6.00 ± 1.00	103.25 ± 1.89^c^	99.75 ± 15.10^b^	0.366 ± 0.017^c^
Diabetic treated (*P. tomentosa*) group	91.00 ± 13.45^x^	44.00 ± 2.64 ^a,x^	8.00 ± 1.00	95.00 ± 0.00 ^b,x^	67.66 ± 18.58^x^	0.340 ± 0.017^c, x^
Treated (*P. tomentosa*) group	93.75 ± 7.08	44.25 ± 4.031	7.66 ± 1.52	76.00 ± 2.00	63.00 ± 9.64	0.187 ± 0.006

Results are presented as mean±SD. n=5. ^a^ *P<*0.01, ^b^ *P<*0.05, and ^c^ *P<*0.001 represented the statistically significant difference between the control and all treated groups. ^x^ *P<*0.01 also showed a significant difference between the diabetic untreated group and the diabetic group treated with *P. tomentosa*

**Table 3:** Retention times (Rt), molecular formula, Δ ppm, [M−H]-, [(M+FA)-H]- and MS/MS values of compounds occurring in the aqueous extract of *Pergularia tomentos*a aerial parts identified by LC-ESI/LTQOrbitrap/MS/MS (negative ion mode)

n	*R*_t_ * (min)	molecular formula	Δ ppm	[(M+FA)-H]_-_	[M-H]^-^	MS/MS	Compound
**1**	9.67	C_30_H_44_O_13_	1.81	611.2687		564, 467, 421	ghalakinoside
**2**	11.07	C_30_H_42_O_12_	-2.04	593.2580		547, 529, 419	6'-hydroxycalactin
**3**	11.70	C_29_H_40_O_11_	-1.86		563.2476	519, 347	12 β ,6'-dihydroxycalotropin.
**4**	11.99	C_24_H_36_O_8_	-1.85	451.2312		405	12 β-hydroxycoroglaucigenin
**5**	12.25	C_30_H_44_O_12_	-1.75	595.2738		549, 387	12'-dehydroxyghalakinoside
**6**	12.35	C_21_H_20_O_12_	-1.69		463.0859	301	quercetin 3-*O*-β-D-galactopyranoside
**7**	12.41	C_30_H_42_O_12_	-1.61	593.2283		547, 419	16α-hydroxycalotropin
**8**	12.61	C_30_H_46_O_12_	-1.86	597.2884		551, 373	glucocoroglaucigenin
**9**	13.03	C_21_H_20_O_11_	1.69		447.0914	285, 255, 227	kaempferol-3-*O*-β-D-galactopyranoside*
**10**	13.39	C_21_H_20_O_11_	1.69		447.0914	285, 255, 227	kaempferol-3-*O*-β-D-glucopyranoside*
**11**	13.69	C_22_H_22_O_12_	-1.41		477.1021	357, 315	isorhamnetin-3-*O*-β-D-glucopyranoside
**12**	14.10	C_30_H_42_O_12_	-1.71	593.2582		529, 419, 401	6'β-hydroxycalotropin
**13**	14.21	C_30_H_42_O_12_	-1.40	593.2584		547, 419	12β-hydroxycalactin
**14**	14.47	C_30_H_44_O_12_	-1.60	595.2740		549	6'-dehydroxyghalakinoside
**15**	14.67	C_35_H_50_O_14_	-1.19	739.3163		693	3-*O*-*β*-glucopyranosiyl-calactin
**16**	15.19	C_30_H_46_O_11_	-1.65	581.2947		535, 391,373	desglucouzarin
**17**	16.02	C_24_H_36_O_7_	-1.63	435.2370		389	coroglaucigenin
**18**	17.04	C_32_H_44_O_13_	-2.09	635.2685		589, 571	16α-acetoxycalotropin
**19**	17.50	C_30_H_42_O_11_	-1.61	577.2634		531, 403, 373,	calotropin
**20**	18.80	C_30_H_42_O_11_	-1.66	577.2634		531, 403, 373, 271	calactin
**21**	20.05	C_39_H_52_O_12_	-2.55		711.3357	696, 571, 373	6'-*O*-feruloyl-desglucouzarin
**22**	21.69	C_34_H_46_O_14_	-2.32	677.2788		631, 571, 461	16α-acetoxyasclepin
**23**	22.46	C_32_H_44_O_12_	-2.48	619.2734		573, 531	asclepin

* low intensity

**Table 4:** Quantitative results of representative compounds occurring in *Pergularia tomentos*a aerial parts

Compound	**MRM transition**	**R** ^2^	**Regression line**	**mg/100 g aerial parts** ^*^ ** ±SD** ^**^
ghalakinoside (**1**)	611→421	0.98	y= 48.9x-1.14e-4	3.54 ± 0.11
6'-hydroxycalactin (**2**)	593→547	0.99	y= 50.5x-1.35e-4	4.95 ± 0.70
12'-dehydroxyghalakinoside (**5**)	595→549	0.99	y= 39.5x+2.83e-5	7.11 ± 0.03
12-hydroxycalactin (**13**)	593→547	0.99	y= 55.3x-2.56e-4	5.58 ± 0.95
6'-dehydroxyghalakinoside (**14**)	595→549	0.99	y= 33.7x+1.74e-5	11.60 ± 0.49
coroglaucigenin (**17**)	435→389	0.99	y= 55.3x-2.56e-4	0.95 ± 0.01
calactin (**20**)	577→531	0.99	y= 46.5x-1.01e-4	2.02 ± 0.44

* dry weight, ** standard deviation

## Conclusion

This is the first report on the evaluation of the hypoglycemic activity of *P. tomentosa* by* in vivo *assays. This study led to highlight the capability of aqueous extract of *P. tomentosa* aerial parts to decrease the glucose levels and induce beneficial effects on lipid profile in STZ-induced diabetic rats. In particular, *P. tomentosa*’s effects on hyperglycemia and hyperlipidemia have been displayed. These effects could be due to antioxidant properties, a decrease in glucose level and triglyceride levels as well as effects on the liver enzymes. Furthermore, to correlate the hypoglycemic activity to the chemical composition of *P. tomentosa*, an analytical approach based on the acquisition of a metabolite profile by LC-ESI/LTQOrbitrap/MS/MS analysis has been carried out. In this way, the identification of cardenolides, along with flavonoids, has been accomplished.

Therefore, a synergism among secondary metabolites, e.g., cardenolides able to inhibit Na^+^/K^+^-ATPase pump and flavonol glycosides exerting antioxidant activity, occurring in the aqueous extract of *P. tomentosa *aerial parts, could be supposed to explain the hypoglycemic effects exerted by *P. tomentosa*.

## Authors’ Contributions

SHH designed the work, analyzed the data, and drafted the manuscript. SP contributed to the design of the work and to writing the manuscript. ARQ and FEG participate in the study design, biological data collection, and manuscript correction. AC performed the LC-MS analysis. All authors read and approved the manuscript. 

## Availability of data and materials

All data generated or analyzed during this survey are included in this article.

## Consent for publication

All authors give consent for the data to be published.

## Conflicts of Interest

All authors declare no conflicts of interest regarding this work. 
